# Tuberculosis in Sulaimaniyah, Iraqi Kurdistan: A Detailed Analysis of Cases Registered in Treatment Centers

**Published:** 2016

**Authors:** Kamaran Karadakhy, Nasih Othman, Faredun Ibrahimm, Akam Ali Saeed, Ari Abdul-Adheem Hama Amin

**Affiliations:** 1 School of Medicine, University of Sulaimani, Sulaimaniyah, Iraq; 2 Kurdistan Institution for Strategic Studies & Scientific Research, Sulaimaniyah, Iraq; 3 Shar Hospital, Sulaimaniyah, Iraq; 4 Sulaimani Teaching Hospital; 5 Center for the Respiratory and Chest Disease Sulaimaniyah, Iraq

**Keywords:** Tuberculosis, DOTS, Incidence, Iraq

## Abstract

**Background:**

Tuberculosis (TB) remains a major public health problem especially in low and middle-income countries. The current study was undertaken to estimate the incidence of the disease and describe its epidemiological characteristics in Iraqi Kurdistan.

**Materials and Methods:**

A retrospective study was carried out on cases registered in the directly observed treatment-short course (DOTS) centers in Sulaimaniyah province. Information was collected from the summary reports of all cases registered in 2010 and detailed information was obtained from 307 cases in the main center.

**Results:**

During 2010, a total of 530 new and relapsed cases were registered in the DOTS centers amounting to an annual incidence of 31 per 100,000. Over 73% of cases were pulmonary TB and 45% of all cases had positive smear. Most common symptoms were cough (58%), sweating (49%) and fever (48). Almost 43% of patients were diagnosed by direct swab examination, 30% by biopsy and 23% through clinical and radiologic examination. In relation to outcome, 89% of patients were treated successfully, 7% died and 3% defaulted. Mortality rate was 8% in pulmonary infection and 4% in extrapulmonary infection. Old age (65 years and over) was significantly associated with higher odds of death compared to people aged 34 years and younger (OR 6.7, 95% CI 1.3–36.1, P=0.03).

**Conclusion:**

The incidence of TB is still high in the Iraqi Kurdistan. The DOTS has been successful in treating the majority of cases but there are areas needing improvement especially record-keeping and patient follow-up during and after treatment.

## INTRODUCTION

Tuberculosis remains a major public health problem especially in low and middle-income countries. According to the latest report by the World Health Organization (WHO), an estimated 9.6 million new and relapsed cases of TB occurred globally of which around 86% were from Asia and Africa (1). This amounts to a global incidence of around 133 per 100,000 per year. According g to the same report, an estimated 1.5 million people died from the disease, the majority being from Africa and south East Asia and about 400,000 being HIV+ (1). The WHO estimate for the incidence in the Eastern Mediterranean region is 117 per 100,000 per year. However, reports from some countries of the region based on patient records report a lower incidence rate. A retrospective study from Saudi Arabia covering reported cases in 20 years (1991–2010) estimates an annual incidence rate of 14 to 17 per 100,000 (2). A recent study from the same country reports a similar incidence of 13.8 per 100,000 in 2011. In Iran, the incidence of TB is estimated at around 12 per 100,000 (3). Community-based studies have reported a higher prevalence of the disease. The prevalence of smear positive TB was 255 per 100,000 in central India (4), 145 in Vietnam (5) and 34 in China (6).

Like other countries of the region, TB has been a major public health issue in Iraq. The national TB control program was started in 1989 and the DOTS was introduced in 1998 which gradually expanded until in 2008 it covered all 18 provinces (7). According to the WHO, Iraq is among the seven countries of the Eastern Mediterranean region with high prevalence of TB accounting for 3% of cases in the region. In 2014, 8,268 new and relapsed cases of TB were reported in the Iraq resulting in an estimated incidence rate of 43 per 100,000 per year with a case detection rate of 54% (8). Another study conducted in 2011 estimates the total number of cases in Iraq to be 14,500 (95% CI 13,200–17,200) with an annual incidence rate of 45 per 100,000 per year (9). The current study was undertaken in Sulaimaniyah province to describe the epidemiology of TB in the province and analyze cases registered at the DOTS centers in relation to demographic characteristics, signs and symptoms and treatment outcomes.

## MATERIALS AND METHODS

Sulaimaniyah is one of the three provinces of Kurdistan Region-Iraq with an estimated population of around 1,700,000 in 2010. There is a main TB center in the province center and four centers in the major districts. Information was collected from the summary reports of all cases reported by all centers during 2010. These summary reports are routinely prepared for surveillance purposes. We used these data to estimate the incidence of TB in the province and provide an overall description of all cases as shown in Tables 1 and 2. In addition to this, detailed information about the cases and their management using DOTS was retrieved from patients’ files for 2010–2011 available at the main center in Sulaimani city. Data were transcribed from files and records of patients into a data collection form and later entered into Epidata (10). These were the only files which were available at the center and accessible to the research team. Patients’ files included data on demographics, the disease, drugs, outcome and contacts. Descriptive analysis was done in Stata version 9 (11). The research did not involve actual patients but only their medical records. Patients’ privacy and confidentiality were respected and the study was approved by the relevant health authorities and the TB center.

**Table 1.** Characteristics of cases registered in the Sulaimaniyah TB canter in 2010 (530 cases)

	**Number**	**%**
**All**	530	100.0
Males	264	49.8
Females	266	50.2
**Type**		
Pulmonary	391	73.8
Extra-pulmonary	139	26.2
**Diagnosis**		
New	512	96.6
Relapse	18	3.4
**Sputum**		
Positive	240	45.3
Negative	290	54.7
**Age group (yrs)**		
1–14	18	3.4
15–24	62	11.7
25–44	233	44.0
55–64	167	31.5
65 and over	46	8.7
**HIV +ve**	0	0.0
**Registration time**		
First quarter 2010	133	25.1
Second quarter 2010	115	21.7
Third quarter 2010	146	27.5
Fourth quarter 2010	136	25.7
**Contacts**		
Sputum samples examined	1689	
Sputum +ve	67	4.0

**Table 2.** Sites of extra-pulmonary tuberculosis, Sulaimaniyah 2010 (139 cases)

	**Number**	**%**
**Lymph nodes**	42	30.2
**Pleura**	27	19.4
**Gastrointestinal tract**	21	15.1
**Genitourinary tract**	14	10.1
**Other**	13	9.4
**Skeleton**	11	7.9
**Meninges**	5	3.6
**Skin**	4	2.9
**Pericardium**	2	1.4
**All**	139	100

## RESULTS

During 2010, a total of 530 patients were registered in the DOTS centers across Sulaimaniyah governorate of which 512 cases (97%) were new cases and 18 cases (3%) were relapsed cases. This results in an estimated annual incidence of 31 per 100,000 per year. Males and females were affected in similar proportions. Over 73% of cases were pulmonary TB and 45% of all cases yielded positive microscopic results in direct sputum examination. The older age groups were more affected with 40% of cases affecting people aged 55 years and older and only 3% affecting children up to 14 years of age. See Table 1 for other characteristics of these cases (Table 1).

Of these 530 patients, 139 cases (26.2%) had extrapulmonary infection. The most common types of these infections affected the lymph nodes (30.2%), followed in order of frequency by plural infection (19.4%) and gastrointestinal tract infections (15.1%). Other organ sites less frequently affected were genitourinary tract, bone, meninges and skin (Table 2).

Files of 307 patients were available for further analysis including 150 males and 157 females. The age of patients ranged from 4 months to 95 years [mean of 50 years, standard deviation (SD) of 22.0 years]. Over 64% of patients had pulmonary TB, 94% were new infections and 48% were positive for sputum examination. Other characteristics of these patients are shown in Table 3.

**Table 3.** Characteristics of 307 patients studied in detail

	Number	%
**All**	307	100
Males	150	48.9
Females	157	51.1
**Residence**		
Sulaimaniyah city	157	51.1
Outside Sulaimaniyah city	120	39.1
Other provinces	24	7.9
Foreign nationals	4	2.9
Missing	2	
**Type**		
Pulmonary	197	64.2
Extra-pulmonary	110	35.8
**Diagnosis**		
New	269	94.1
Relapse	18	5.9
**Sputum**		
Positive	98	48.0
Negative	106	52.0
Missing	103	
**Referral from**		
Public hospital	57	35.4
Private hospital/clinic	103	62.8
Self	1	1.8
Missing	144	
**BCG scar present**	302	99.0
**Family income**		
Good	106	79.1
Not good/bad	28	20.1
Missing	173	
**Education**		
None	66	59.5
Basic	28	25.2
High school and higher	17	15.3
Missing	196	
**Occupation/role**		
Housewife	93	30.8
Child/student	56	18.5
Old/retired	54	17.9
Private sector	51	16.9
Public servant	31	10.2
Prison mate	13	4.3
Foreign worker	4	1.3
**Outcome**		
Recovery	118	59.3
Treatment completed	59	29.7
Death	14	7.0
Default	6	3.0

### Signs and symptoms

The most common symptoms are shown in Table 4. Cough was the most common symptom which happened in 58% of patients with a mean duration of 10.5 weeks (SD: 10.7) followed by sweating which happened in 49% of patients with a mean duration of 8 weeks (SD: 9.3). Other common symptoms were fever, fatigue and weight loss (Table 4). Over 11% of patients reported previous history of TB. The history of previous TB dated back to 1–30 years ago with a mean of 8.3 years (SD: 10 years).

**Table 4.** Signs and symptoms in order of frequency

**Symptom/sign**	**Yes Number (%)**	**No Number (%)**	**Mean duration in weeks (range, standard deviation)**
**Cough**	92 (58)	67 (42)	10.5 (2–50, 10.7)
**Sweating**	78 (49)	81 (51)	8.0 (1–50, 9.3)
**Fever**	77 (48)	82 (52)	7.4 (1–50, 8.6)
**Fatigue**	76 (48)	83 (52)	8.5 (2–50, 9.6)
**Weight loss**	73 (46)	85 (54)	9.0 (2–50, 8.2)
**Sputum**	67 (42)	92 (58)	11.3 (1–50, 13.8)
**Chest pain**	64 (40)	95 (60)	-
**Past history if TB**	18 (11.5)	139 (88.5)	8. 3 years (1–30, 8.9)

The mean family size of patients was 5.4 (SD: 2.6) ranging from 1–14 persons. Therefore, there was an average of 4.4 contacts per patient. Family history of TB was positive in 11% of patients with 80% of families having one person affected and 12% of patients having two family members with history of TB. The overall prevalence of smoking amongst patients was 11% (males 20%, females 3%, P=0.001). Eight percent of the patients were diabetic.

### Diagnosis

Table 5 shows results of some diagnostic procedures performed for all patients. Almost 43% of patients were diagnosed by direct swab examination, 30% by biopsy and 23% based on symptoms detected via clinical and radiographic examinations. The first sputum examination which was done at registration was positive in 48% of patients and subsequent examinations done in one month intervals were mostly negative. The plain chest X-rays of cases diagnosed with pulmonary TB showed positive lesions in the right lung (47% of cases), left lung (31%), and both lungs (14%) while in 8% of cases lungs were clear. None of the patients showed positive results for hepatitis B or HIV.

**Table 5.** Diagnostic procedures

**Method of diagnosis at registration**		
Direct swab	42.7%
Biopsy	30.4%
X ray	12.9%
Symptoms	9.9%
Culture	4.1%
**Sputum examination positive**		
Sputum first sample	48.1%
Sputum second	4.7%
Third	1.0%
Fourth	1.5%
**Radiologic findings**		
Right lung lesions	35.7%
Left lung lesions	20.4%
Both lungs affected	9.4%
Lungs clear	34.5%
**ESR at registration**		
Mean (SD)	33.8 mm (30)
Range	2–114 mm

### Treatment

In phase one, 274 patients (91%) were treated with ethambutol, pyrazinamide, rifampicin and isoniazid, 3% with the above-mentioned combination in addition to streptomycin, 3% with rifampicin and isoniazid and a few patients with other combinations. The mean duration of phase one was 2.1 months (SD 0.9). In phase two, 224 patients (86%) were treated with rifampicin and isoniazid, 5% with the two drugs in addition to ethambutol and 4% with the three drugs plus pyrazinamide. The mean duration of phase 2 was 4.5 months (SD 2.1). In phase one, 18% of patients had at least one day interruption of treatment with a mean of 2.7 days (SD 10 days). While in phase 2, the interruption happened in 30% of patients with a mean of 1.7 days (SD 7 days). In both phases combined, 38% of patients had at least on day interruption.

### Outcome

In relation to outcome of treatment, 59% of patients were reported as having recovered, 30% as having completed treatment, 7% died and 3% defaulted. Table 6 shows mortality by different characteristics. Death was significantly associated with age. While 15% of patients aged 65 years and over died, only 2% of those aged 0–34 and 8% of those aged 35–64 years died (χ^2^ =7.5, P=0.03). Death was also significantly associated with negative sputum results where mortality was 16% in sputum negative cases compared to 3% in positive cases (χ^2^ =8.9, P=0.01). Mortality rate was 8% in pulmonary infections and 4% in extrapulmonary infections but the difference was not statistically significant. Mortality was similar in 2010 and 2011 and in both sexes. When these factors where entered into a logistic regression model only old age remained statistically significant. The odds ratio for death in the elderly aged over 65 years was 6.7 compared to the younger patients aged 0–34 years (95% CI 1.3–36.1, P=0.03).

**Table 6.** Mortality based on different characteristics of patients

**Characteristic**	**% died**	**P value**
**Total**	7.0	
Male	5.3	
Female	8.5	0.37
**Age group**		
0–34 years	2.3	
35–64 years	7.9	0.03
65 and over	15.4	
**Type**		
Pulmonary	8.4	
Extra-pulmonary	4.3	0.28
**Year**		
2010	7.1	
2011	6.9	0.99
**Sputum**		
Positive	2.9	
Negative	16.3	0.01
	103	
**Missed days of treatment**		
Yes	8.6	
No	6.1	0.5

## DISCUSSION

### Key findings

The incidence of TB in the province was 31 per 100,000 per year with similar number of cases in males and females. The age of patients ranged from 4 months to 95 years (mean 50 years). Over 64% of patients had pulmonary TB, 94% had new infections and 48% were positive for sputum examination. The most common symptoms were cough (58%), sweating (49%) and fever (48%). Over 11% of patients reported previous history of TB. Radiographic findings were present in 92% of pulmonary cases with right lungs more frequently affected (61% of cases).The DOTS regimen included ethambutol, pyrazinamide, rifampicin and isoniazid in 91% of cases. The mean duration of phase one was 2.1 months and the mean duration of phase 2 was 4.5 months (SD: 2.1). Death was significantly associated with age with an odds ratio of 6.7 in the elderly aged 65 years and over compared to the younger patients aged 0–34 years.

### Limitations of the study

This study analyzed cases registered at the DOTS centers; thus, it inherited the limitations of routine data such as missing values, inconsistency, and incompleteness. Certain information recorded in the files comes from patients telling the history of their disease; therefore, recall bias could not be ruled out. Estimate of incidence is based on cases registered at the centers; thus, it could be an underestimation of true incidence.

### Comparison with other studies

The DOTS strategy is built on five key components for its successful implementation which are political commitment, case detection with availability of lab facilities, standardized treatment with supervision, effective drug supply and management system and regular monitoring and evaluation (12). Our results showed that centers followed the two phases of treatment (2+4 months) as recommended by the WHO; over 80% of patients in phase one and 70% in phase 2 had no interruptions in treatment and interruptions were very short with a mean of two days. While this could be an indication of good compliance, it only reflects the time lag reported to centers and does not mean that all drugs have been taken as recommended. Although DOTS recommends that, at least during the initial phase of treatment, patients have to be directly observed swallowing the tablets, in practice, this is difficult to follow. The usual practice is that patients are first given medications for 2 weeks and if adherence is good and no side effects are reported, the patient is requested to show up once a month to collect medications for the next month. However, if compliance is not good or the patient fails to visit the center as scheduled, designated staff from the center will actively follow the case. In such circumstances and in either case it is hard to verify the strict compliance with the regimen by all patients.

A study from Iran (13) reports several factors for patient compliance to DOTS regimens including personal beliefs about the disease, privacy concerns, health staff role and other social factors. In terms of drug supply, we noticed that there was no shortage of drugs during the time period, and all patients were given the required drugs according to the regimen during their visits. However, in relation to patient records, we noticed incompleteness and inconsistency in patient information. For example, files of 2010 were more completely filled compared to those of 2011. In the latter, some fields such as patient symptoms had up to 50% missing values. This could be due to changes in management and health staff and lack of regular monitoring which is one of the pillars of DOTS.

According to our study, the incidence of all registered TB cases in 2011was 31 per 100,000 (of which 14 per 100,000 were smear- positive). In the same year, the expected WHO estimate for Iraq was 45 per 100,000 (9). Comparing these two estimates and assuming the same expected incidence for Sulaimaniyah province, the case detection rate would be 69%. Incidence of all forms of TB in the same year was less in Saudis (10 per 100,000) according to Memish et al (14). Our incidence rate was also higher than that of Iran (12 per 100,000 in 2015) as reported by Moosazadeh et al (3). However, rates are much higher in south Asia such as India (4), Vietnam (5) and china (6).

In our study, extrapulmonary infections comprised 26% of cases and the most common extrapulmonary infections involved lymph nodes, pleura and gastrointestinal tract. A study from Germany reports extrapulmonary manifestations in 22% with more cases in females, children and persons originating from Africa and Asia (15). A study from India reports extrapulmonary infection in 15–20% of all cases of TB and 50% of HIV-positive individuals with lymph nodes being the most common site of involvement (16). Another study from the United States also reports lymphadenitis as the most common type of extrapulmonary TB followed by skeletal TB in about 35% of cases. This study reported up to 50% extrapulmonary involvement in patients with concurrent AIDS and TB (17). The risk of extrapulmonary TB appears to be more in immunocompromised patients.

In relation to signs and symptoms, the most common were cough, fever, fatigue and weight loss. The classic symptoms used in the simple case definition of TB (cough, fever and weight loss) were present in 40% of all cases and 48% of cases with pulmonary TB. Other studies have shown variable proportions of signs and symptoms in pulmonary and extrapulmonary TB but cough is usually the most common presenting symptom in all studies. A study from China reported that 48% of newly diagnosed TB patients were asymptomatic and about 50% of them did not report cough of any duration (18). According to a study from Turkey, the most common symptoms were cough (55%), weakness (53%) and night sweats (45%) (19). However, these symptoms are related to the severity and type of the disease at the time of diagnosis where many pathological and sociocultural factors could be related to late presentations (19).

Some symptoms of TB are similar to simple respiratory infections and may cause delay in seeking care in addition to sociocultural factors. A study from Croatia reports the most common reason for delay in seeking care and diagnosis of TB to be mistaking the symptoms for influenza in over one-third of patients and that almost 30% of TB patients remain undiagnosed for over 30 days after the initial health care visit (20).

In our study, 13% of cases were diagnosed through radiography and 10% symptomatically. We observed that the TB center staffs were under some perceived pressure to rise up to the WHO recommendations for a higher case detection rate and probably this might have influenced favorable diagnosis of suspected cases. The new diagnostic platform, the GeneXpert OmniR, has been developed for point-of-care testing for TB and rifampicin-resistance using existing Xpert MTB/RIFR cartridges (1). This facility was not available for use during the current study. Laboratory confirmation of TB and testing for drug resistance are essential to make sure suspected cases are correctly diagnosed and have access to the appropriate treatment as soon as possible. However, this is still not widely possible in many countries where clinical diagnosis still remains acceptable. The WHO report estimates that out of 5.2 million new and relapsed pulmonary TB patients in 2014, only 3 million (58%) were bacteriologically confirmed (1).

Around 89% of patients in the current study either recovered or completed treatment (successful treatment), 3% defaulted and 7% died. It has to be noted that the mortality rate calculated here was actually the case fatality rate during the follow-up time which was generally 8 months of treatment. Some studies have calculated survival or death rates per 100,000 persons/year during extended periods of time which would be different. Our mortality results are comparable to those of a study from Uzbekistan (21) reporting 83% successful treatment rate, 3% default and 6% mortality rate but more than those of a study from Turkey with 93% cure or complete treatment, 4% default and 2.4% mortality (22). A study from Ethiopia reports a higher death rate of 9%, successful treatment rate of 60% and a default rate of 9% (23). Another study reports 4% mortality (24). The mortality estimates of TB patients could differ because of type of cases included, varying follow-up times, poor follow-up information and presence of comorbidities.

## CONCLUSION

The study showed a high incidence of TB in the region compared to the surrounding countries. The DOTS strategy has been successful in treating around 90% of registered cases successfully but there are areas needing improvement. Follow-up of patients during and after treatment needs more attention. Patient records need extensive work in order to make sure that data are collected more consistently and completely and kept for future use. Digitalization of patient records is essential to prevent loss of records as the files are routinely destroyed after several years. Regular monitoring of work of the DOTS centers and training of their staff are essential parts of a successful DOTS strategy implementation. Further research, including qualitative, is required to probe into the causes of compliance and patient attitudes and behaviors with regard to treatment.
